# Case Report: Nicolau syndrome due to etofenamate injection

**DOI:** 10.12688/f1000research.11705.1

**Published:** 2017-06-12

**Authors:** Emin Ozlu, Aysegul Baykan, Ragıp Ertas, Yılmaz Ulas, Kemal Ozyurt, Atıl Avcı, Halit Baykan

**Affiliations:** 1 Department of Dermatology, School of Medicine, Duzce University, Duzce, 81620, Turkey; 2 Department of Dermatology, Kayseri Tekden Hospital, Kayseri, 38000, Turkey; 3 Department of Dermatology, Kayseri Training and Research Hospital, Kayseri, 38010, Turkey; 4 Department of Plastic and Reconstructive Surgery, Kayseri Training and Research Hospital, Kayseri, 38010, Turkey

**Keywords:** Complication, etofenamate, Nicolau syndrome

## Abstract

Nicolau syndrome, also known as embolia cutis medicomentosa, is a rare complication characterized by tissue necrosis that occurs after injection of drugs. The exact pathogenesis is uncertain, but there are several hypotheses, including direct damage to the end artery and cytotoxic effects of the drug. Severe pain in the immediate postinjection period and purplish discoloration of the skin with reticulate pigmentary pattern is characteristic of this syndrome. Diagnosis is mainly clinical and there is no standard treatment for the disease. Etofenamate is a non-steroidal anti-inflammatory drug and a non-selective cyclooxygenase inhibitor. Cutaneous adverse findings caused by etofenamate are uncommon. Herein, we present a case with diagnosis of Nicolau syndrome due to etofenamate injection, which is a rare occurrence.

## Introduction

Nicolau syndrome is a rare complication caused by intramuscular injection of various medications ^1^. The necrosis in the injection site of skin and sometimes muscle is a characteristic feature of this syndrome ^1^. The development of acute vasospasm following intravenous or around the vein injection is the most widely accepted hypothesis in its pathogenesis ^1^. Etofenamate is an anti-inflammatory drug that non-selectively inhibits the cyclooxygenase (COX) pathway ^2^. Herein, we present a rare case of Nicolau syndrome after etofenamate injection.

## Case report

**Figure 1.**
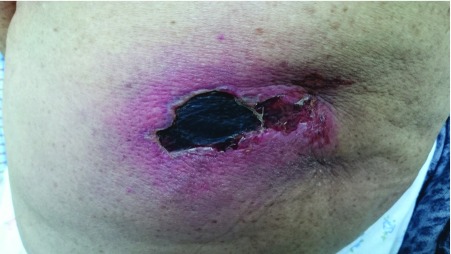
Black, necrotic ulcerated plaque on the gluteal region 15 days following etofenamate injection.

An 81-year-old woman was admitted to our clinic with a painful necrotic ulcer in the left gluteal region. Her medical history, which was non-specific, except for back pain, revealed an intramuscular etofenamate injection (1000 mg), due to back pain, 15 days before. Dermatological examination revealed a painful ulcerous plaque with a black necrotic crest in the lateral part of the left gluteal region. This ulcerous plaque appeared indurated and erythematous in its surrounding ( Figure 1). Her complaints started with erythematous swelling and pain in the injection site approximately ten days ago. Subsequently, the ulcer developed in the lesion area of the patient's erythematous swelling. There were not any abnormal parameters in both complete blood count and routine biochemistry tests. The patient was diagnosed with Nicolau syndrome based on her medical history and clinical signs and symptoms. Biopsy from the lesion area was not obtained, as it could develop more necrosis in the lesion. Etofenamate treatment was discontinued.

**Figure 2.**
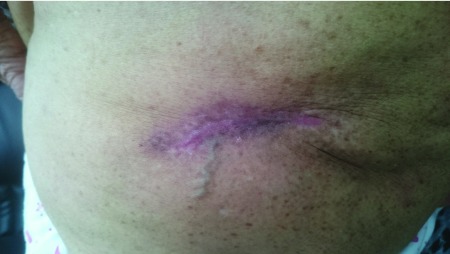
Large atrophic, deppressed scar on the gluteal region one month following treament.

Local wound care with saline solution once a day and topical 2% mupirocin twice a day was applied to the lesion and the patient was referred to the Department of Plastic Surgery for the debridement of the necrotic tissue. After surgical debridement by the plastic surgeon, and continuation of local wound care (as above), the ulcer lesion was completely regressed, leaving an atrophic scar after one month ( Figure 2).

## Discussion

Nicolau syndrome, also known as embolia cutis medicamentosa, is defined as an iatrogenic syndrome following intramuscular injections. However, cases with Nicolau syndrome after subcutaneous, intravenous, or intraarticular injection have been recently reported in the literature ^3– 5^.

Although the pathogenesis of Nicolau syndrome is not fully understood, direct vascular damage, perivascular inflammation, and vascular contraction following an injection are thought to be responsible ^6^. In addition, it has been suggested that pharmacological properties of an individual drug may play a role in the pathogenesis ^6^.

Etofenamate is a non-steroidal anti-inflammatory drug (NSAID) with analgesic, antipyretic, and anti-inflammatory effects. It inhibits the COX pathway and blocks prostaglandin synthesis non-selectively ^2^. It has been shown that NSAIDs play a key role in the pathogenesis of vascular spasm induction and local circulation blockage, inhibiting the COX enzyme and prostaglandin synthesis ^7^. In addition, these drugs have a central role in inducing vascular spasm and blocking local circulation, inhibiting the COX enzyme and prostaglandin synthesis in the pathogenesis of this syndrome ^7^.

In Nicolau syndrome, following the injection of the clinically active agent, erythematous, ecchymosed, and reticular lesions appear in the injection site with severe pain. Progressive ischemic necrosis with sharp edges in a livedoid pattern develops later. Lesions often heal leaving atrophic scars ^8^.

Nicolau syndrome has no definitive treatment. In the early period, the main goal of therapy is to prevent the development of necrosis. Therefore, pentoxifylline, hyperbaric oxygen, intravenous alprostadil, and heparin, which strengthen the vasculature, can be used ^4^. Intralesional steroid injection can also be effective by reducing inflammation. Surgical debridement should be performed in the case of necrosis ^4^. Systemic antibiotics should be used in case of secondary infection ^4^. Contraction and deformity development are among late complications, and surgical treatment can be required in these cases ^9^. Nicolau syndrome is uncommon with proper injection techniques - aspirating just before injecting medication has been suggested as a technique of preventing this syndrome ^10^.

## Conclusion

As a result, applications of standard drug injection rules are essential in prevention from Nicolau syndrome. It should be kept in mind that Nicolau syndrome could also develop following the use of intramuscular etofenamate.

## Consent

Written informed consent was obtained from the patient for the publication of the patient’s clinical details and accompanying images.
