# Supplementary material for: Case Report: Nicolau syndrome due to etofenamate injection
Source: F1000Res. 2017 Jun 12;6:867. [Version 1] doi: 10.12688/f1000research.11705.1 (PMC5558102; doi:10.12688/f1000research.11705.1)
Supplement: Peer review history

# Peer review: Referee response for version 1

**DOI:** 10.5256/f1000research.12646.r24881

**Published:** 2017-08-14

**Authors:** Burak Tekin

**Affiliations:** 1 Private dermatology specialist, Istanbul, Turkey

Well-presented case of Nicolau syndrome.

 Dermatologists seem to be familiar with this entity, however, all healthcare workers may encounter this reaction in their clinical practice since injectable NSAIDs are commonly used. This report may serve the purpose of increasing awareness with regard to this entity, while placing emphasis on the importance of adhering to the proper injection technique.

I have read this submission. I believe that I have an appropriate level of expertise to confirm that it is of an acceptable scientific standard.

# Peer review: Referee response for version 1

**DOI:** 10.5256/f1000research.12646.r23434

**Published:** 2017-07-03

**Authors:** Mahmut Sami Metin

**Affiliations:** 1 Department of Dermatology, Faculty of Medicine, Atatürk University, Erzurum, Turkey

- The article has been well designed.
- Pictures are good.
- Discussion is long enough.
- The quality of the research is good enough.
- The work has been well designed, executed and discussed.
- No changes are required.
- The authors could use this new research article " Nicolau Syndrome due to Penicillin Injection: A Report of 3 Cases without Long-Term Complication." 

I have read this submission. I believe that I have an appropriate level of expertise to confirm that it is of an acceptable scientific standard.
